# The complex landscape of TMS devices: A brief overview

**DOI:** 10.1371/journal.pone.0292733

**Published:** 2023-11-28

**Authors:** Ane Miren Gutiérrez-Muto, Sven Bestmann, Rubén Sánchez de la Torre, José L. Pons, Antonio Oliviero, Jesús Tornero

**Affiliations:** 1 Center for Clinical Neuroscience, Hospital Los Madroños, Brunete, Madrid, Spain; 2 Department of Clinical and Movement Neurosciences, UCL Queen Square Institute of Neurology, University College London, London, United Kingdom; 3 Wellcome Centre for Human Neuroimaging, UCL Queen Square Institute of Neurology, University College London, London, United Kingdom; 4 Legs and Walking Lab, Shirley Ryan Ability Laboratory (Formerly Rehabilitation Institute of Chicago), Chicago, IL, United States of America; 5 Department of Physical Medicine and Rehabilitation, Feinberg School of Medicine, Northwestern University, Chicago, IL, United States of America; 6 Advanced Neurorehabilitation Unit, Hospital Los Madroños, Brunete, Madrid, Spain; University of Catania Libraries and Documentation Centre: Universita degli Studi di Catania, ITALY

## Abstract

The increasing application of TMS in research and therapy has spawned an ever-growing number of commercial and non-commercial TMS devices and technology development. New CE-marked devices appear at a rate of approximately one every two years, with new FDA-approved application of TMS occurring at a similar rate. With the resulting complex landscape of TMS devices and their application, accessible information about the technological characteristics of the TMS devices, such as the type of their circuitry, their pulse characteristics, or permitted protocols would be beneficial. We here present an overview and open access database summarizing key features and applications of available commercial and non-commercial TMS devices (http://www.tmsbase.info). This may guide comparison and decision making about the use of these devices. A bibliometric analysis was performed by identifying commercial and non-commercial TMS devices from which a comprehensive database was created summarizing their publicly available characteristics, both from a technical and clinical point of view. In this document, we introduce both the commercial devices and prototypes found in the literature. The technical specifications that unify these devices are briefly analysed in two separate tables: power electronics, waveform, protocols, and coil types. In the prototype TMS systems, the proposed innovations are focused on improving the treatment regarding the patient: noise cancellation, controllable parameters, and multiple stimulation. This analysis shows that the landscape of TMS is becoming increasingly fragmented, with new devices appearing ever more frequently. The review provided here can support development of benchmarking frameworks and comparison between TMS systems, inform the choice of TMS platforms for specific research and therapeutic applications, and guide future technology development for neuromodulation devices. This standardisation strategy will allow a better end-user choice, with an impact on the TMS manufacturing industry and a homogenisation of patient samples in multi-centre clinical studies. As an open access repository, we envisage the database to grow along with the dynamic development of TMS devices and applications through community-lead curation.

## Introduction

Transcranial magnetic stimulation (TMS) is a non-invasive brain stimulation technology used for basic science, translational research, and therapy in a wide range of neurological and psychiatric disorders [1–3]. The technological evolution of TMS has greatly facilitated the insights into human brain function and the development of novel diagnostic and therapeutic tools [4–7].

In recent years, increased regulatory control has been achieved to respond to the increase in the number and complexity of TMS systems. The International Organisation for Standardisation (ISO) is widely accepted at international level and provides governments with a technical basis for health, safety, and environmental requirements [8], and TMS devices are designed on ISO principles. Generally, every country has its own market regulations. However, the European Union, due to the number of countries it integrates, and the FDA, due to its impact on the market, are the most recognized worldwide. The FDA only applies in the United States, but from the industrial perspective it has a great impact on scientific societies and end users.

The ISO 14971 defines risk management in the regulation of medical devices. Furthermore, ISO 13485 states that it is necessary to ensure that design and testing of the device are sufficient and effective, and that manufacturers uniformly meet the requirements for the establishment and maintenance of a quality management system. These principles are codified and harmonized by FDA in the Quality System Regulation (QSR) [9]. For TMS devices, manufacturers must identify safety measures and reduce every possible risk. The IEC establishes globally accepted guidelines to ensure the safety of electrical products [10]. Medical devices are specifically subject to the IEC-60601 family of standards, most importantly the general safety controls in IEC-60601-1, where the electromagnetic compatibility of devices is defined [11]. To the best of our knowledge, all commercial TMS devices adhere to internationally accepted standards.

Although all commercial equipment is compliant with the legal framework of the country of use, compliance with FDA certification procedures (in the USA) and CE marking (in the EU) are the norm, due to the size of the respective markets. Out of the commercially available stimulators we here identified, most are FDA-cleared and all of them are CE marked [12]. In addition to the full and adequate description of the TMS stimulus, aspects related to the reliability of the device, including safety and performance controls, must comply with several technical standards. However, no standards specific to the manufacture of TMS devices have been developed. Ideally, over time, a particular unified and harmonized standard for TMS devices (or non-invasive brain stimulation devices more generally) would emerge, along with a benchmarking framework, to be used alongside standards reflected in documents such as certificates of conformity, FDA regulations, quality certificates, etc. that are already in use [13, 14].

Within the set of TMS research prototypes, we can point out the existence of proposals for the development of home-made devices in nonclinical environments for self-improvement purposes [15, 16].

### Commercial TMS systems and prototype devices

**Fig 1:**
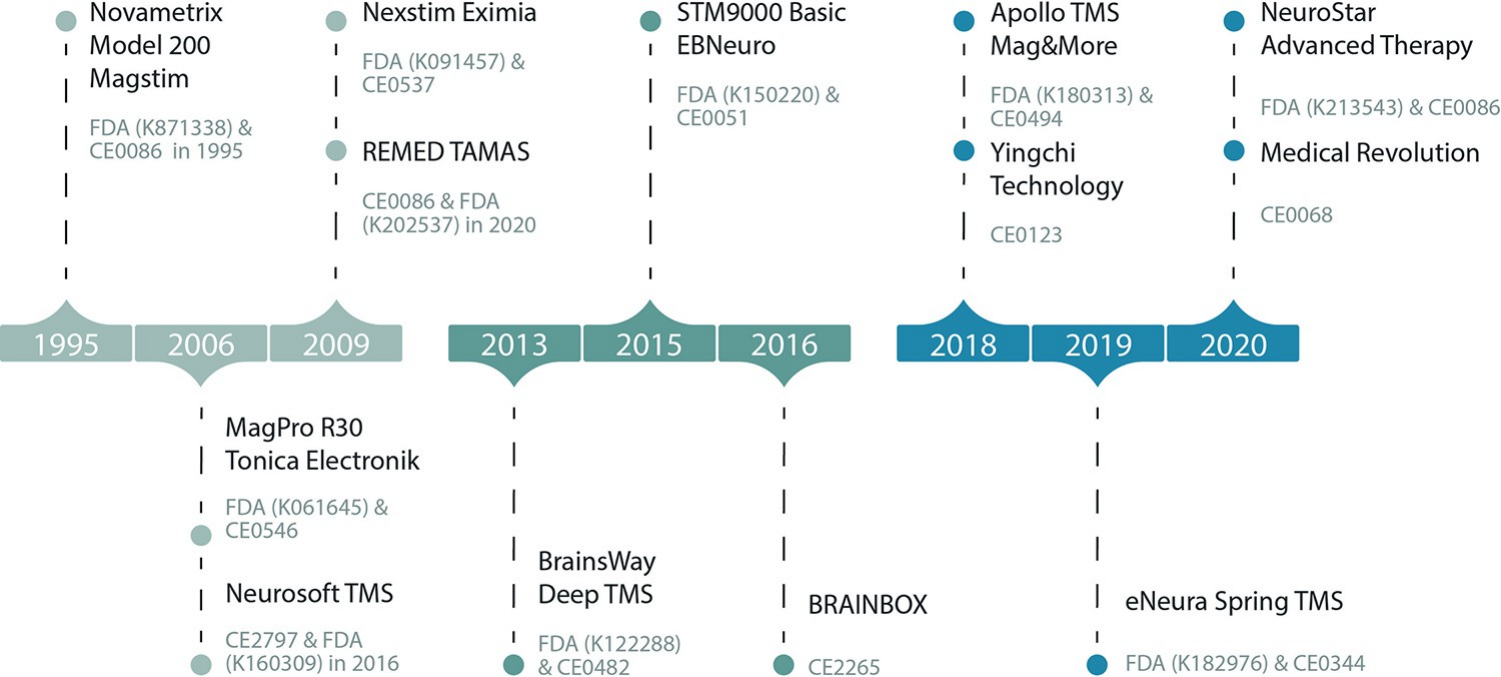
TMS device timeline. Certification timeline for the different TMS trademarks (first time certificated).

There are now several commercial TMS systems that have entered the market (see Fig 1), with varying degrees of transparency about their technical details and information available that is independent of the manufacturer. We note that several devices are marketed under more than one name: For example, CloudTMS (K173441) and Soterix Medical MEGA-TMS (K192823) correspond to the Neurosoft Neuro-MSD device, and the Deymed devices are marketed also by Brainbox Ltd. There is also the case of collaborations between different manufacturers: for example, Yingchi Techology’s "Hanix" device is the Brainsway system marketed in China.

**Fig 2:**
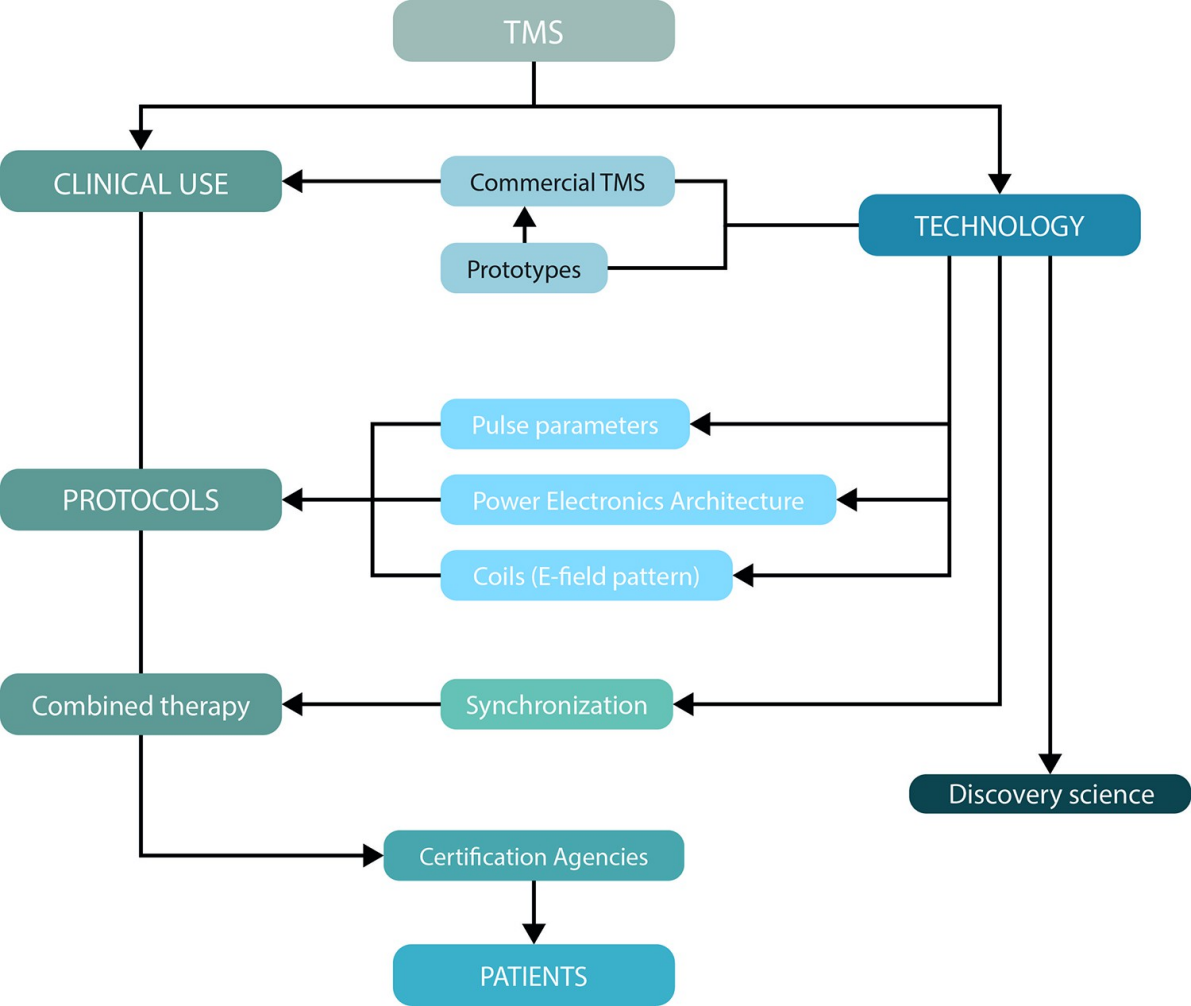
Workflow. Applications that drive TMS device development.

The development of TMS devices is generally motivated by the clinical application of TMS with a requirement for patient-friendly usage [17], or development for discovery science which often requires technological development and prototypes for novel research questions [18, 19] (see Fig 2).

For example, newly developed experimental devices now allow more extensive control over the pulse characteristics compared to clinical TMS devices [20, 21]. Such new developments are generally used for research first, but the technology may subsequently transfer to clinical TMS applications, with several examples of TMS prototypes being commercialised [22, 23].

### TMS technology

The power circuit of a TMS unit consists of a high voltage (HV) supply (voltages in the order of 2000V and capable of supplying currents over 5000A), a HV capacitor which acts as an energy storage (and able to supply capacitance values of around 180 μF and voltages in the range of 1-3kV), a power switch (usually diodes or thyristors), and a stimulation coil. All these components must be designed to operate under high voltages and high currents. Existing commercial devices use the basic configuration in the pulse switch [24], i.e., a circuit based on thyristors and diodes (Silicon-Controlled Rectifiers, SCR technology) with less turn-off capability that allows current control through the thyristor and higher pulse current rating. Insulate Gate Bipolar Transistors (IGTBs) or Metal-Oxide-Semiconductor Field-Effect Transistors (MOSFETs) are also used in the latest technology [25, 26]. These fully controllable switches can alter the voltage pulses and are easier to use due to their simple and fast turn-off behavior [27]. To the extent that such information is available, our database summarizes key technological features of different devices, but we encourage the field to further contribute such information where it is known.

Two different classes of stimulus waveforms can be distinguished: biphasic or monophasic [28, 29], although recent developments also allow for more flexible configuration of TMS pulse shapes [30, 31]. Ultimately, this waveform depends on the designed power electronic architecture based on IGBT, thyristors, or diodes-thyristors [20].

For repetitive stimulation protocols, stimulation frequencies are commonly in the range of 1 to 100Hz [32]. The technology sets the limits to the range of stimulation parameters including pulse intensity, frequency, and pulse width [33, 34]. As a rule of thumb, monophasic waveforms do not permit high frequency stimulation, whereas stimulation intensity may be limited for high stimulation frequencies, owing to the power bottlenecks created by such protocols [35]. Pulse shapes determine the physiological impact of TMS, emphasizing the relevance of summarizing the technical specifications of TMS devices. Concerning the shape of the coils, the majority of TMS coils are circular or figure-of-eight shaped, with a range of sizes [36]. For the purposes of this database, we did not review the shape and models of the coils due to the large number of coils and their varied applications [37, 38].

We here present an open access database (http://www.tmsbase.info) with key features and application of available commercial and non-commercial TMS devices, based on an review of the information available. The database (TMS platform) can help to address the need for defining standard parameters and technical determinants of stimulation characteristics of TMS devices. Moreover, this database can help researchers and clinicians compare the information provided from manufacturers [39] and select devices and protocols [40], that address their specific needs.

## Methods: Source of data and search strategy

A bibliometric analysis was performed by identifying TMS manufacturers globally to create a comprehensive database (http://www.tmsbase.info). The goal is to provide a systematic overview of the different commercial TMS devices available on the international market, as well as their most important technical and clinical characteristics, and the emerging trends TMS experimental devices.

**Fig 3:**
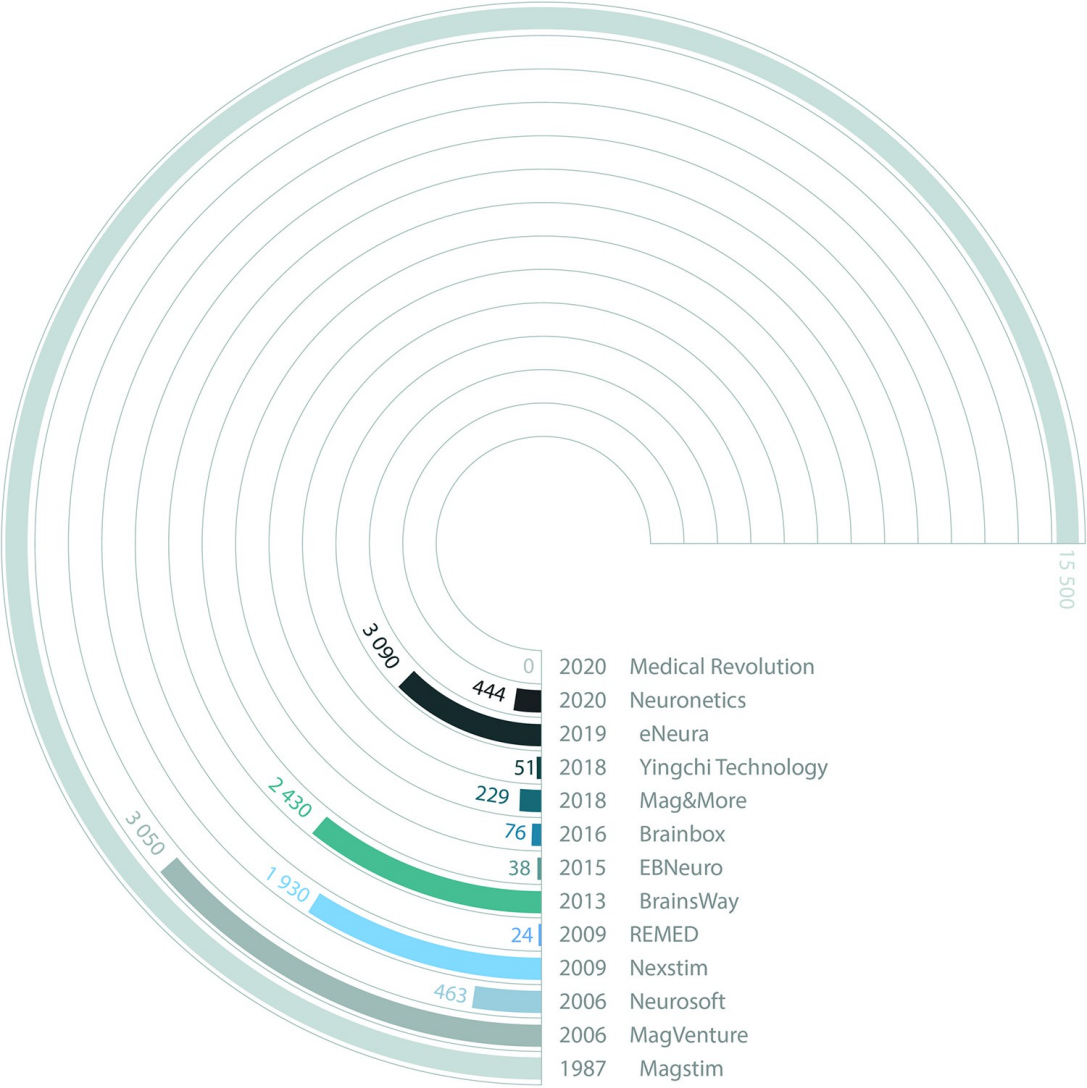
Research information. Number of published articles for each commercial device shown in Fig 1.

Fig 3 shows the number of scientific publications and studies related involving commercial TMS devices to date (Fig 1). The first commercial systems have been used extensively for research and are currently the most widely used devices in the clinical environment. However, the latest commercial equipment to reach the market is also starting to be used in research applications.

For the commercial devices, we performed the literature search on Google Patents and Google Scholar databases, using the commercial name of the stimulation device followed by the word "TMS". The total result of the search procedure is shown in Fig 3. Since the number of articles related to the first commercial TMS devices is very high, for this work we have considered as a priority the studies provided for each commercial device in the last few years. In addition, a Google search was performed with the words "Transcranial Magnetic Stimulation device" for a more accurate result. We include as a source of information each user manual with technical specifications and other technical data. Additional information about each of the commercial devices can be found in the websites of each company. We note that some information will inevitably not be accessible due to being proprietary or not being disclosed by companies. However, we would like to highlight that all the information included in the manuscript is public and from open access repositories. Information provided in company manuals or websites may be incomplete or only provide estimates for user guidance. Such information should therefore be taken with a pinch of salt when attempting a detailed technical appraisal of each device, and where possible, we have indicated the source of the information in the table. However, the database also affords the opportunity to update and complete this information in the future.

For the experimental (non-commercial) TMS devices we performed the literature research on Google Scholar database with the following search terms: “transcranial magnetic stimulation”, “TMS”, “circuit”, “technology”, “topology”, “components” and “coils”. A record was considered relevant if included a circuit topology explanation or if a detailed coil development was enclosed. A total of 283 articles and patents were searched, of which only 117 were relevant to this study according to the criteria mentioned above. We applied no further criteria for selecting the articles.

## Results

### Commercial TMS devices

In the following, we summarize some of the key features common to many commercial TMS devices (for further information access the web page with the database). Table 1 presents the thirteen commercial TMS manufacturers on the market. Of the thirteen manufacturers, six include families with at least five different devices. There are two manufacturers that include three different systems in their portfolio and only one manufacturer in the market includes two systems. The remaining four manufacturers offer only one TMS system.

**Table 1:** Commercial TMS devices.

	Device family	Pulse mode and protocols	Frequency (up to)	Pulse-width and waveform type	Thyristors and/or diodes	IGBT and/or MOSFET	External trigger connection mode	Coil refrigeration	Sham coil	Magnetic field (max. Tesla)	Ref.
**Magstim**	Magstim 200^2^	Single-pulse (spTMS)	Up to 0.5Hz	Monophasic: 80μs	✓		RS232 Connector and CMOS logic level	Air-cooled coils	✓	>1.44T	[28, 49]
	Magstim BiStim^2^	Paired-pulse (ppTMS)		Monophasic							
	Magstim Rapid^2^	spTMS, repetitive (rTMS)	15 Hz at 100% MSO	Biphasic: 350μs							
	Magstim Super Rapid^2^	spTMS, rTMS, Theta Burst protocol (TBS)	Up to 100 Hz. 25 Hz at 100% MSO	Biphasic							
	Magstim Super Rapid^2^ Plus	spTMS, rTMS, TBS	Up to 100 Hz. 41 Hz at 100% MSO	Biphasic							
**MagVenture**	MagPro R20/R20+/express	spTMS, rTMS	Up to 20 Hz (R20+ or R20+ Express solution up to 100Hz)	Biphasic	✓		DSUB9 and BNC connectors and TTL level	Active cooling (fluid)	✓	2T	[50]
	MagPro R30 w/n MagOption	spTMS, rTMS, TBS, dual up to 5Hz, twin pulses	Up to 30Hz (also 60Hz and 80Hz solutions)	Monophasic: 70μs, biphasic: 280μs							
	MagPro R100	spTMS, rTMS		Biphasic							
	MagProX100 w/n MagOption	spTMS, rTMS, TBS, half-sine, twin and dual up to 5Hz, power pulse (40% increase)	Up to 100Hz	Monophasic: 70–100μs, biphasic: 280–400μs							
	XP Orange edition	spTMS, rTMS, TBS	Up to 250Hz	Biphasic							
	MagPro Compact	spTMS, rTMS, TBS	Up to 5Hz	Biphasic							
**Nexstim**	Stand-alone TMS	spTMS, rTMS, ppTMS (monophasic)	Up to 10 Hz at 50% MSO, 4 Hz at 100% MSO		✓		TTL and CMOS levels	-	unknown	199V/m	
	SmartFocus TMS		Up to 8 Hz at 50% MSO, 3.3 Hz at 100% MSO	Monophasic, biphasic: 230μs							
**BrainsWay**	Deep TMS	rTMS		Biphasic: 400μs	unknown		-	-	-	unknown	
**EBNeuro**	STM9000	spTMS, rTMS, TBS, double stimulation function (BI option)	Basic: up to 10Hz Standard: up to 30Hz Fast: up to 50Hz Ultra-fast: up to 100Hz	Monophasic and biphasic	unknown		TTL level	Air-cooled coils	✓	4.2T	
**Brainbox**	DuoMag MP	spTMS, rTMS, TBS	Up to 2Hz, 0.5Hz at 100% MSO, 1.4Hz at 50% MSO	Monophasic: 100μs		✓	BNC connector and TTL level	Active liquid cooling and air-cooled coils	✓	700J	[51]
	DuoMag MP-Dual	spTMS, ppTMS	Single pulse: up to 4Hz, 1Hz at 100% MSO, 2.8Hz at 50% MSO. Paired pulse: up to 2Hz, 0.5Hz at 100% MSO, 1.4Hz at 50% MSO	Monophasic: 100–120μs							
	DuoMag MP-Quad	spTMS, ppTMS, quadri-pulse Theta Burst (qTBS)	Single pulse: up to 8Hz, 2Hz at 100% MSO, 5.6Hz at 50% MSO. Paired pulse: up to 4Hz, 1Hz at 100% MSO, 2.8Hz at 50% MSO. Quadripulse: up to 2Hz, 0.5Hz at 100% MSO, 1.4Hz at 50% MSO								
	DuoMag XT-10	spTMS, rTMS	Up to 10Hz, 5Hz at 100% MSO, 10Hz at 50% MSO	Biphasic: 290μs							
	DuoMag XT-35	spTMS, rTMS	Up to 35Hz, 5Hz at 100% MSO, 35Hz at 50% MSO								
	DuoMag XT-100	spTMS, rTMS	Up to 100Hz, 22Hz at 100% MSO, 86Hz at 50% MSO								
	Elevate TMS	spTMS, ppTMS, qTBS, rTMS TBS, controllable (cTMS)	Up to 1kHz	Variable up to 385μs							
**Neurosoft**	Neuro-MSX Advanced Therapeutic	rTMS, ramp, sweep frequency	Up to 100Hz (2kHz for burst mode), 35Hz at 100% MSO	Biphasic: 250–330μs	✓		-	✓	✓	unknown	[52]
	Neuro-MSX Advanced	rTMS, ramp, sweep frequency	Up to 100Hz, 15Hz at 100% MSO								
	Neuro-MS/D Therapeutic	rTMS	5Hz at 100% MSO, 20Hz with power supply	Biphasic: 250–330μs							
	Neuro-MS/D Advanced Therapeutic	rTMS	Up to 100Hz, 20Hz at 100% MSO								
	Neuro-MS Diagnostic Monophasic	spTMS, ppTMS, configurations for triple- or quadri- pulse (QPS) stimulation.	0.3Hz at 100% MSO	Monophasic							
	Neuro-MS Diagnostic Paired-Pulse										
**Mag&More**	Apollo PowerMAG Research 30	spTMS, rTMS, ramp	Up to 30Hz	Monophasic: 80μs, biphasic: 160μs		✓	BNC connector and TTL level	✓	✓	4T	
	Apollo PowerMAG Research 100	spTMS, rTMS, ramp, TBS	Up to 100 Hz at 70% MSO, 50 Hz at 80% MSO and 30 Hz at 100% MSO								
	Apollo PowerMAG Research ppTMS	spTMS, ppTMS, rTMS, ramp, TBS		Biphasic: 160μs							
	Apollo PowerMAG Research MR	spTMS, rTMS, ramp, TBS									
	Apollo PowerMAG multifocal			Monophasic: 80μs, biphasic: 160μs							
	Apollo PowerMAG QPS		100Hz (specific qTBS protocol at 666Hz in bursts)								
**Yingchi Technology**	M-10 Ultimate	spTMS, rTMS	Up to 10Hz		✓		COM PORT and TTL level	Liquid and air-cooled coils	✓	>1T	[53]
	M-30 Ultimate		Up to 30Hz								
	M-50 Ultimate	spTMS, rTMS, TBS	Up to 50Hz								
	M-100 Ultimate		Up to 100Hz								
	E series	spTMS, rTMS, TBS, ramp	Up to 100Hz, 35Hz at 100% MSO								
**Neuronetics**	NeuroStar	rTMS	Repetitive up to 30 Hz, burst up to 20 Hz	Biphasic: 185μs	✓		BNC, USB, ethernet and RS232 connector	-	✓	0.5T	[54]
**eNeura**	Spring TMS	spTMS		A rise time of 180μs and a total pulse length of less than 1ms	unknown		-	-	-	1.3T	[55]
**Medical Revolution**	MagRex	spTMS, rTMS	Up to 150Hz	Biphasic: 300μs	unknown		-	-		7.5T	
	Neuro-MS		Up to 30Hz	Biphasic: 250μs							
	Neuro-MSL										
**REMED**	ALTMS (TAMAS)	spTMS, rTMS, ppTMS	Up to 15 Hz at 100% MSO, 20 Hz at 80% MSO, 30 Hz at 50% MSO and 50Hz at 20% MSO	Biphasic: 350μs	unknown		D-type connector and TTL level	-		3T	
	ALTMS-A	spTMS, rTMS	Up to 100Hz	Biphasic: 450μs							
	Brain-Stim-A (portable)										

Comparison of the different technical aspects of commercial TMS devices.

### Power electronics architecture

Table 1 shows a total of 47 different devices from thirteen manufacturers. The architecture of the stimulation circuitry in these devices is based on parallel assembly of thyristors and diodes depending on the stimulation pulse waveform. Regarding the power electronic circuit, and as shown in Table 1, half of the devices include thyristors and diodes, two devices include new power components such as IGBTs or MOSFETs as a result of the advances in semiconductor technology. For four devices we could not identify information on the topology of their power electronics.

### Pulse shapes

As shown in Table 1, fifteen of the commercial devices that we identified have the option of monophasic pulses. It shows twenty-nine systems that can be identified as biphasic and three systems that could not be classified with respect to their waveform. Of the fifteen monophasic, only one has a pulse width of 70μs, while other five devices have a pulse width of 80μs. Three systems offer the possibility of varying the pulse width from 70μs to 120μs and no information is available for the remaining devices. As mentioned above, twenty-nine devices have biphasic waveform option. Seven devices work with pulses below 200μs and there are twelve biphasic TMS systems with time widths ranging from 200μs to 350μs. Finally, two systems operate with pulse widths above 400μs and only one system offers the possibility of varying the pulse width in a temporal range between 280μs and 400μs. For the remaining seven biphasic devices we have not found any information available.

### Stimulation coils

Although commercial TMS systems offer a wide variety of patient coils, their basic geometry is very similar [41, 42]. However, the strategy for coil heat dissipation varies between the different systems, and includes internal air venting, refrigerant flow cooling or passive refrigerant cooling [35]. The different types of coil cooling can also modify the conditions under which the stimulation can be delivered [43, 44]. Several systems also have optional sham coils [6, 45, 46] that offer scalp and sound stimulation without effective stimulation of cortex, with near identical appearances to active coils.

### Stimulation protocols and output intensity

Several stimulation patterns and protocols [47] are available for commercial devices, broadly falling within these categories: single pulses, paired pulses, repetitive pulses [2], and patterned repetitive protocols [48]. All of these have been evaluated and work under the support of the guidelines and recommendations to prevent adverse effects during the application of TMS in research and clinical settings [47].

Magnetic field strength is perhaps one of the parameters that best characterizes commercial TMS systems. Unfortunately, each manufacturer identifies the field strength differently. Some manufacturers provide the maximum magnetic field strength, which varies from 0.5T to 7.5T, while others indicate the energy delivered to the patient per pulse in Joules or the maximum electric field strength in V/m. Another parameter that characterizes TMS systems is the pulse rate. Most commercial systems offer maximum pulse frequencies between 10Hz up to 100Hz. However, as can be seen in Table 1, there are two systems that achieve pulse frequencies of 150Hz and 200Hz. In addition, there are two manufacturers that, upon customer’s request, can provide systems with pulse frequencies of that reach 1kHz and 2kHz respectively. Currently, controllable pulses (cTMS), in which the amplitude, width and shape of the pulse can be modified, are only available for one device on the market.

### Commercial TMS lifetime

The average lifetime of TMS devices is determined both by a limited number of years–this varies between 5 and 10 years depending on the manufacturer–and by the number of pulses generated by the system (between 10^6^ and 2x10^7^ discharges, which depends on the lifetime of the capacitor). The stimulation coils also have their own lifetime depending on the number of discharges. Manufacturers also provide information on connection requirements and power consumption, as well as applied standards for electromagnetic compatibility.

### Research prototypes

**Fig 4:**
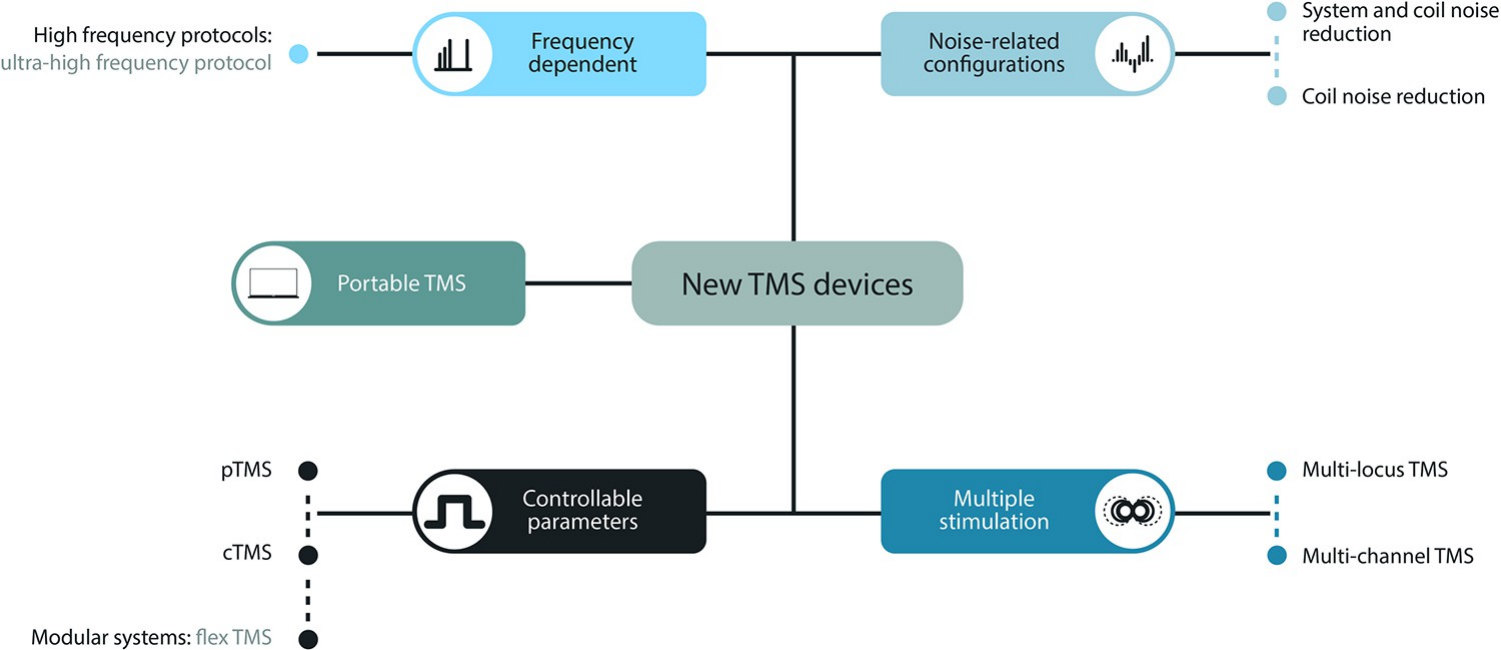
Workflow of the classification of TMS research systems.

Fig 4 shows an overview of the workflow leading to classification of new TMS systems. The prototypes found in the literature are classified according to the objective of each of the systems. We here distinguish five different categories (see Table 2): i) portable TMS devices ii) ultra-high frequency devices iii) controllable, programmable, and modular systems, iv) noise reduction systems, and v) multiple-stimulation devices.

**Table 2:** Classification of research TMS devices.

Research systems: categories/subcategories		Objective	Technical characteristics	Ref.
Portable TMS		A portable prototype (sTMS mini) for use in clinical trials to manage the pain associated with migraine with Aura.	Standard thyristor configuration with 100μs pulse duration and a circular coil.	[58, 81–83]
Frequency dependent				
	Ultra-high frequency burst protocol	Plasticity induction of a novel protocol that merged theta burst stimulation (TBS) and quadri-pulse stimulation (QPS	A high voltage power supply with voltage power IGBT.	[48, 84, 85]
Controllable parameters				
	pTMS	Two generator system with programmable stimulus pulses and patterns.	Pulse-width modulation with a non-resonant, high-frequency switching architecture based on an H-bridge inverter (with IGBTs).	[31, 68, 86, 87]
	cTMS	Three generators of controllable pulse parameter devices.	Two terminals of stimulation coil driven by two half-bridge circuits with current-bidirectional switches implemented by IGBT modules.	[23, 88]
	Modular systems			
	*flexTMS*	Magnetic pulses which can be adjusted.	Full-bridge circuit incorporating 4 IGBT modules and 1 energy storage capacitor.	[89, 90]
		Generate a nearly arbitrary output voltage waveform.	Cascade H-bridge inverter topology with ten modules. Each one using low-voltage MOSFET.	[27, 91]
		Flexibly generate high-power TMS pulses with user-defined electric-field shape.	Modular circuit topology and unipolar field-effect silicon-carbide (SiC) transistors.	[30, 92, 93]
		Medical application and analysis of a multilevel stimulator.	Each module has eight low-voltage MOSFET operated in parallel.	[70]
Noise-related configurations				
	System and coil noise reduction			
		Reduction of the coil current pulse duration and redesign the mechanical structure of the stimulation coil.	A circular coil that suppresses the conversion of electrical into acoustic energy	[71, 94, 95]
		A quiet TMS with ultra-short rectangular pulses and an increased peak voltage, and a coil designed electrically and mechanically to minimize the emitted sound.	An H-bridge circuit with IGBTs is implemented for ultra-brief pulses, with a circular coil	[75, 95]
		An active noise control (ANC) strategy with online identification, offline analysis, and real-time output.	ANC strategy can effectively reduce the coil noise synchronously after the first stimulation sequence and in the subsequent treatment.	[78]
	Coil noise reduction			
		A quiet coil design technique where the Lorentz self-force is optimized to reduce the acoustic noise.	Rectangular flat coils, spherical, and hemispherical coils.	[96, 97]
		A stimulation coil with reduced sound emissions.	A stimulation coil with at least one coil winding core and one double casing.	[77]
Multiple stimulation				
	Multi-channel TMS			
		Stimulation system based on multi-channel reconfigurable coils to stimulate multiple brain sites in any temporal order.	A wire-mesh coil in *x* and *y* directions and electrically insulated.	[98, 99]
		Simultaneous and sequential operation of five independently controllable channel stimulator.	Five independent channels with a SCR switch and a diode in parallel.	[22, 100]
		Achieve multi-point synchronous stimulation and flexible switching of various stimulation patterns.	Eight discharge circuits controlled by IGBTs and a 4x4 straight wire array with a thick copper plate in it.	[101–103]
	Multi-locus TMS (multi-coil)			
		Overcome the limitations of physical coil movement by introducing electronic targeting.	A set of five resemble coils.	[104]
		Automatically find the optimal stimulation parameters, location, and orientation of an electronically adjusted multi-locus TMS.	Two different transducer configurations	[105–107]
		Multi-loci and multi-site current patterning (sequential or simultaneous) for precise, rapid, and repeatable neuronal targeting.	Three overlapping triangular coil arrays allowing any spatial current distribution.	[80, 108]
		Relation between the number of coils, the focality of the induced electric-field, and the extent of the cortical region that can be controlled.	Five different coil multi-locus, with two different cortical targets.	[79]
		A TMS coil for manipulating the electric-field orientation electronically with high accuracy.	A pair of orthogonally oriented figure-of-eight coils with a minimum-energy optimization procedure.	[79, 106, 107, 109, 110]
		A modular 3-axis TMS coil used in multi-channel TMS systems.	Three orthogonal separate solenoid coils of 8 to 12 turns with an air-cooling system.	[111–114]

### Portable TMS device

The possibility of portable TMS systems is attractive for the treatment of any pathology, and a number of portable devices have been developed and marketed [56–58]. For example, the eNeura device is characterised by single-pulse stimulation (sTMS), with a stimulation frequency of very low Hertz: 1 stimulation pulse every 4 to 15 minutes [59, 60]. This makes it easy to optimise the weight, volume of the system and its components.

### Ultra-high frequency devices

Other devices have been developed for delivery of bespoke high frequency protocols, such as the quadri-pulse theta-burst stimulation (qTBS) protocol. This protocol consists of four single-sine-wave pulses which are given at an ultra-high pulse repetition rate of 200Hz or 666Hz, and a burst repetition rate of 5Hz [48]. In this type of device, a high voltage power supply charges the energy storage capacitor constantly and so the discharging takes less than half of the charging time. To reach these frequencies, power switches in the circuit allow the release of stimuli very close to each other and with short oscillation periods. This protocol has been introduced to the market by companies such as Mag&More and Brainbox, with the stimulators PowerMAG QPS [61] and DuoMAG MP-Quad [62] respectively.

### Controllable, programmable, and modular systems

TMS devices focused on programmable pulse waveforms, where pulse width, intensity, polarity, and inter-stimuli intervals [63–65] can generate flexible pulse waveforms such as consecutive rectangular pulses with a predetermined time interval, width, and polarity. Therefore, these devices are suitable for a range of applications, e.g., analysis of the stimulated neurons and their activation characteristics [66].

Advances in power electronics technology have led to more precise control of the power required in TMS devices [67]. However, existing technology has limitations regarding the ability to control the shape of the generated electrical stimulus [68]. Thus, TMS devices have been divided into independent modules of lower intensity [69], capable of dynamically modifying the output voltage and covering the working range of a conventional stimulator [70].

### Noise reduction systems

The sound generated by a TMS device at the time of treatment has negative effects on the patient, both on their auditory system and by activating regions of the brain involuntarily [71–73]. Studies focusing on noise reduction have examined three different solutions for noise reduction. Firstly, the pulse width reduction, with dominant spectral power above 20kHz, has advantages in the acoustic domain because high frequencies are easier to suppress, and human perception is notably reduced [74, 75]. However, due to the neural membrane time constant, the amplitude of the pulses (hence the peak coil voltage) must be increased to achieve neural stimulation [76]. Secondly, a proper assembly of winding core and casing are proposed to reduce mechanical deformation in the casing, and thus the noise [77]. A phase-shifted elastic coupling between the winding and the coil housing is normally used. In addition, a rigid, frictional viscoelastic layer covering the winding block increases acoustic energy dissipation [75]. Finally, external active noise cancellation strategies are proposed, whereby acoustic sounds (noise) generated by the TMS device are recorded in real-time, and the coil noise is reduced synchronously after the first stimulation sequence [78]. This method reduces the coil noise synchronously after the first stimulation sequence and in the subsequent treatment protocol.

### Multiple-stimulation devices

Multi-channel systems consist of a unique stimulator design with multiple coils, each one controlled by a specific channel and with independent control [22]. By contrast, multi-locus systems consist of the arrangement of different coil patterns, placed in a stack configuration, for the stimulation of brain neighbouring areas and the generation of diverse shapes of electric fields [79, 80]. Some of the coil winding patterns encountered are a figure-of-eight coil and a matched four-leaf-cloves coil, a figure-of-eight coil overlapped with an oval coil, and three overlapping triangular coil arrays among others.

New developments of multi-coil systems now provide the possibility to stimulate several brain regions simultaneously with one device and a single coil [22]. This allows the different stimulation coils to be activated electronically, without the need for physical triggering by the clinician [79]. Moreover, multi-locus systems can control the location and orientation of the peak of the induced electric field within a wider cortical region.

## Discussion

Advances in TMS technology can spawn more efficient stimulation protocols for basic research and clinical applications. Several reviews include evidence-based guidelines on the therapeutic use of rTMS that comprise not only a historical context of the technology, but also its principles and mechanisms of action [2, 32]. Other reviews provide overviews about new stimulation coils, new pulse sequences and novel waveforms for stimulation, with an emphasis on safety recommendations, with updates on training, ethical and regulatory issues [47] or a bibliometric analysis revealing the publication patterns and emerging trends of rTMS [5].

From our analysis it becomes clear that there is substantial variation to which degree different TMS devices will deliver such protocols, and it is largely unclear to what extent such variation may affect the efficacy and physiological consequences of TMS. In the absence of standardization and homogeneity between different commercial TMS devices, the stimulation parameters are not the same, as discussed previously. For example, in the case of the Theta-Burst protocol, there is a trade-off between frequency and maximum stimulation intensity, with a strong dependence on the TMS model used with a direct impact on the effectiveness of the therapy [33].

However, it is well established that pulse-shape is a key determinant of the physiological impact of TMS, and our overview helps assessment of device differences and how these may impact on the efficacy of TMS. Recent devices, for example, allow for flexible control of the stimulation waveform, with the possibility for more rectangular pulses and continuous control of parameters such as pulse width and the positive/negative phase amplitude ratio of the pulse [113]. With the recent development of flexible systems with pulse parameter control, development of future TMS devices is likely to exploit the use of variable stimulus waveforms further, with our database serving as springboard for their comparison.

A recent systematic review includes a summary of the design, positioning, modelling, and optimization of experimental coils with a taxonomic approach [19], with further work emphasizing pulse source technology in coils and TMS-compatible external systems [115]. Coils for deep brain TMS use complex multi-coil designs [116], and multi-channel excitation systems seek to improve focality of stimulation [117]. With this rapidly developing landscape of TMS devices, and no agreed benchmarking framework in place, there is a growing need for detailed synthesis of important technical commonalities and differences of TMS devices.

The lack of standardization and the high variability in the technical characteristics of TMS systems (including pulse duration and shape, output intensity, coil designs, power reductions at high intensity and frequency, etc.) make it difficult to replicate conditions in studies when using a different device. This hampers the search for individualized therapy for clinicians, prescribers, and researchers, and making all this information available can facilitate cross-device comparison, development of common standards, but also the development of novel TMS technology. In the framework of this review, we identify the possible sources of variability in the effects of TMS technique from a technological point of view, by focusing on the device features. However, we have not considered other external sources that could impact on the variability of the TMS effects as procedures [118], application of reference values [119], or experience of the operators [120].

Our overview includes both commercial and custom TMS devices, thus covering a gap that with a comprehensive analysis of technical characteristics not provided elsewhere. The approach with which we summarize TMS technology allows for a broader view of the current status of currently available systems.

## Conclusion

A comprehensive review of both commercial and experimental technology used in transcranial magnetic stimulation systems has been developed. This study includes an open access database (http://www.tmsbase.info) which allows clinicians, prescribers, and researchers to consult the technical characteristics of the equipment and include new systems that they identify of interest to the community. The database will be accessible on a hosing service from a web page, and it will have a contact option where the user will be able to provide information about new devices in a formulary with the TMS that appear in the market.

We here show the latest developments and applications in TMS technology as well as current trends in TMS architecture. The absence of standardization and high variability in the parameter metrics of these systems prevents comparison between different devices, making it difficult for clinicians, prescribers, and researchers to make the right election. Thus, a benchmarking scheme as an initial seed for future international standardization would be of interest to researchers and clinicians as a tool for comparison between TMS systems. To inform the choice of TMS platforms for specific research and therapeutic applications and guide future technology development for neuromodulation devices.
